# Glucose Transporter 8 (GLUT8) Mediates Fructose-induced *de Novo* Lipogenesis and Macrosteatosis*

**DOI:** 10.1074/jbc.M113.527002

**Published:** 2014-02-11

**Authors:** Brian J. DeBosch, Zhouji Chen, Jessica L. Saben, Brian N. Finck, Kelle H. Moley

**Affiliations:** From the Departments of ‡ Pediatrics,; § Medicine, and; ¶ Obstetrics and Gynecology Washington University School of Medicine, St. Louis, Missouri 63110

**Keywords:** Diabetes, Fructose Metabolism, Glucose Transport, Hepatocyte, Metabolic Syndrome

## Abstract

Non-alcoholic fatty liver disease (NAFLD) is the most common liver disease in the world, and it is thought to be the hepatic manifestation of the metabolic syndrome. Excess dietary fructose causes both metabolic syndrome and NAFLD in rodents and humans, but the pathogenic mechanisms of fructose-induced metabolic syndrome and NAFLD are poorly understood. GLUT8 (Slc2A8) is a facilitative glucose and fructose transporter that is highly expressed in liver, heart, and other oxidative tissues. We previously demonstrated that female mice lacking GLUT8 exhibit impaired first-pass hepatic fructose metabolism, suggesting that fructose transport into the hepatocyte, the primary site of fructose metabolism, is in part mediated by GLUT8. Here, we tested the hypothesis that GLUT8 is required for hepatocyte fructose uptake and for the development of fructose-induced NAFLD. We demonstrate that GLUT8 is a cell surface-localized transporter and that GLUT8 overexpression or GLUT8 shRNA-mediated gene silencing significantly induces and blocks radiolabeled fructose uptake in cultured hepatocytes. We further show diminished fructose uptake and *de novo* lipogenesis in fructose-challenged GLUT8-deficient hepatocytes. Finally, livers from long term high-fructose diet-fed GLUT8-deficient mice exhibited attenuated fructose-induced hepatic triglyceride and cholesterol accumulation without changes in hepatocyte insulin-stimulated Akt phosphorylation. GLUT8 is thus essential for hepatocyte fructose transport and fructose-induced macrosteatosis. Fructose delivery across the hepatocyte membrane is thus a proximal, modifiable disease mechanism that may be exploited to prevent NAFLD.

## Teaser

**Background:** GLUT8 is a facilitative fructose and glucose transporter expressed in liver.

**Results:** GLUT8-deficient mice are resistant to fructose-induced fatty liver disease.

**Conclusion:** Hexose transporters can mediate fructose-induced fatty liver disease.

**Significance:** Hepatic hexose transporters represent a novel class of targets to prevent or modulate non-alcoholic fatty liver disease.

## Introduction

Non-alcoholic fatty liver disease (NAFLD)^2^ is the most common form of liver disease in industrialized nations, and it is widely considered to be the hepatic manifestation of the metabolic syndrome (1, 2). Not only is NAFLD a precursor to liver failure and cirrhosis, NAFLD independently predicts development of type 2 diabetes mellitus and cardiovascular disease mortality in men and women (3, 4). The obesity and metabolic syndrome pandemics in Western nations are tightly associated with increased dietary fructose consumption (1); thus, the mechanisms of fructose-induced metabolic syndrome, hepatic steatosis, and progression to non-alcoholic steatohepatitis and cirrhosis are subjects of intense study.

Multiple mechanisms have been proposed to explain NAFLD pathogenesis, including increased dietary fat intake, increased hepatocyte free fatty acid influx, increased *de novo* lipogenesis, impaired fatty acid efflux, and impaired fatty acid β-oxidation (2–5). Epidemiological and agricultural evidence demonstrating the potency of excess dietary carbohydrate in triggering intrahepatic lipid accumulation (steatosis) has long existed, and this has been confirmed in experimental rodent models. Although the stimulatory effect of intracellular carbohydrate on *de novo* lipogenesis is well known (6–10), few data address whether carbohydrate delivery across the hepatocyte plasma membrane *per se* contributes to hepatic steatosis.

In the post-prandial state, carbohydrates enter the hepatocyte from the portal circulation via specific hexose transporters in the glucose transporter (GLUT) family of hexose transporter homologs, including GLUT1, GLUT2, and GLUT5. Of these, GLUT1 is a glucose-specific transporter, GLUT2 transports both glucose and fructose, and GLUT5 transports exclusively fructose (11). Subsequent evidence also demonstrated the dual-specificity glucose and fructose transporter, GLUT8, is also expressed in hepatocytes (12, 13). The contribution of GLUT8 in hepatocyte fructose metabolism and fructose-induced steatosis, however, is unknown.

We recently demonstrated that female mice lacking *SLC2A8* (the gene encoding GLUT8 protein; GLUT8-deficient mice are herein referenced as “GLUT8KO” mice) exhibit increased enterocyte fructose absorption and increased serum fructose concentrations after oral fructose administration (14), suggesting that GLUT8KO mice had impaired hepatic first-pass fructose metabolism. We therefore hypothesized that GLUT8 is required for normal hepatocyte fructose uptake and for fructose-induced hepatic steatosis. Here, we show that GLUT8 is a plasma membrane-localized hepatic transporter and that GLUT8 is rate-limiting for hepatic fructose uptake. Accordingly, GLUT8-deficient hepatocytes exhibited impaired fructose-induced *de novo* lipogenesis and were resistant to high-fructose diet (HFrD)-induced hepatic macrosteatosis and triglyceride accumulation. We conclude that GLUT8 mediates hepatic fructose uptake and HFrD-induced macrosteatosis *in vivo* and that substrate delivery mechanisms at the hepatocyte cell surface are a mechanism by which to modulate NAFLD pathogenesis.

## EXPERIMENTAL PROCEDURES

### GLUT Transcript Quantification

cDNA generated from primary mouse hepatocytes (15) or isolated villous enterocytes (16) was subjected to quantitative real-time PCR. Absolute quantitation was carried out using cDNA standards and primer sets (kindly provided by Paul Hruz, Washington University School of Medicine) as described (17).

RNA-seq data for human liver tissue was acquired in FASTQ format from the Illumina BodyMap project (version 2.0) (18). Alignment to the human genome (hg19) was carried out using Bowtie (18). All data were analyzed in Avadis-NGS and SeqMonk software packages. Uniquely aligned reads were quantified in Avadis-NGS and gene level reads per kilobase per million mapped reads values were calculated.

### Immunofluorescence and Confocal Microscopy

Confocal microscopy was performed on methanol-fixed frozen liver sections or on HepG2 cell cultures using an Olympus Fluoview FV500 laser scanning confocal microscope as described (13, 14). All images were scanned sequentially to minimize fluorescent signal crossover in co-localization experiments. Na^+^/K^+^ ATPase (catalogue no. SC-16041, Santa Cruz Biotechnology, Santa Cruz, CA), transferrin receptor (catalogue no. 13–6890, Invitrogen), and GLUT8 (13, 14) antibodies used for immunofluorescence were incubated (1:250) at 4 °C overnight prior to incubation with anti-goat, anti-mouse, or anti-rabbit (all from Santa Cruz Biotechnology) fluorescent conjugate secondary antibody. DNA was stained with TOPRO-3 iodide (Invitrogen). Co-localization in a minimum of 5–10 confocal images obtained from three distinct wild-type mouse livers or from three distinct HepG2 cultures was quantified. Pearson correlation coefficients for probe co-localization were calculated by ImageJ analysis software (version 1.47) using the JACoP co-localization plug-in (19).

### HepG2 Cell Culture and Fructose Uptake Assay

HepG2 human hepatoblastoma cells were obtained directly from the American Tissue Culture Center and maintained in standard growth medium per ATCC specifications.

### Fructose Uptake in HepG2 Cells

Prior to assay, HepG2 cultures were transfected with adenovirus encoding GFP or GLUT8 (Applied Biological Materials, Richmond, BC) or with lentivirus encoding scrambled or GLUT8-directed shRNA (14) (Sigma) in growth medium containing 0.5% serum overnight. On the day of assay, cultures were deprived of intracellular hexoses for 10 min in Hanks' balanced salt solution (HBSS, Invitrogen). The medium was then replaced with HBSS containing 1 μCi/ml [^14^C]-d-fructose. After 4 min, HepG2 cells were quenched and washed in ice-cold PBS and lysed in 0.1 n NaOH and 1% SDS. 80% of each lysate was subjected to liquid scintillation counting. Protein content in the remaining lysate was quantified by BCA assay (Pierce) according to the manufacturer's instructions. Uptake was normalized for protein content.

### Mice

GLUT8KO mice were generated as described (18). Age-matched female mice were analyzed in all studies described (129SV background). A 60% fructose diet (Harlan Teklad, Madison, WI) was administered *ad libitum* to female mice for 10 days, 4 weeks, or 24 weeks as described (13, 14). Food consumed was measured weekly by food weight throughout the dietary intervention. Mice were fasted overnight prior to sacrifice. All animal procedures were approved by the Washington University School of Medicine Animal Studies Committee.

### Western Blot Analysis

Western blotting was performed as described previously (21). The following antibodies (1:1000 dilution) were used as described: phospho- and total AKT (21), GLUT1 (22), GLUT2 and GLUT5 (13. 14), GLUT8 (13, 14, 23), and GLUT9 (24).

### Primary Mouse Hepatocyte Studies

#### 

##### Determination of Lipogenesis and Oxidation Rates and Fructose Uptake

Primary mouse hepatocytes were obtained as described (15). Radiolabeled triglyceride and fatty acid synthesis was measured as reported (15, 25), using uniformly radiolabeled [^14^C]-d-fructose (American Radiolabeled Chemicals Inc, St. Louis, MO) as substrate. Briefly, after overnight culture in 10% FBS in DMEM, hepatocytes were washed with PBS and cultured in serum-free DMEM for 1 h, and then incubated with fresh DMEM containing 1.5 μCi/ml labeled [^14^C]d-fructose for 2.5 h. Thereafter, the cells were harvested for determination of cellular contents of radiolabeled triacylglycerol (20) or the [^14^C]-labeled fatty acids after saponification with a 30% KOH-70% alcohol solution and extraction with petroleum ether as described (25). Rates of lipogenesis were expressed as disintegrations per minute/mg protein/h. Fat oxidation rates were quantified precisely as described (15, 25).

##### Primary Hepatocyte Fructose Uptake Measurements

Mouse hepatocytes were cultured overnight in 10% FBS in DMEM and deprived of intracellular hexose with HBSS for 10 min in HBSS. The medium was then replaced with HBSS containing 1 μCi/ml [^14^C]d-fructose. After 1 min, hepatocytes were quenched and washed in ice-cold PBS and lysed in 0.1 n NaOH and 1% sodium dodecyl sulfate. 70% of each lysate was subjected to liquid scintillation counting, and the remaining lysate was quantified for protein content by BCA assay. Uptake was normalized to protein content.

### Quantitative Real-Time RT-PCR

Quantitative real-time RT-PCR (qRT-PCR) was performed as reported previously (13, 14) with some modifications. Dissected livers were snap-frozen and then homogenized in TRIzol reagent (Invitrogen). RNA isolated according to the manufacturer's protocol was reverse-transcribed using the Quantitect Qiagen reverse transcriptase kit (Qiagen, Valencia, CA). cDNA was subjected to quantitative PCR using the SYBR Green master mix reagent (Applied Biosystems, Carlsbad, CA). Primers used are listed in Table 1 (13, 26, 27).

**TABLE 1:** **Primers used in real-time quantitative RT-PCR** Sequences are listed 5′-3′.

Target	Forward primer	Reverse primer	Ref.
**ACC1**	TGTCCGCACTGACTGTAACCA	TGCTCCGCACAGATTCTTCA	27
**ChREBP**	CTGGGGACCTAAACAGGAGC	GAAGCCACCCTATAGCTCCC	13
**FAS**	CCTGGATAGCATTCCGAACCT	AGCACATCTCGAAGGCTACACA	27
**SREBP1**	CCAGAGGGTGAGCCTGACAA	AGCCTCTGCAATTTCCAGATCT	27
**PPARα**	TGGTTCCTGGTGCCGATTTA	ACTAGCATCCCACTTAATTATGTATCTGAA	27
**LXRα**	CGACAGAGCTTCGTCCACAA	GCTCGTTCCCCAGCATTTT	27
**FXR**	CACGAAGATCAGATTGCTTTGC	CCGCCGAACGAAGAAACA	27
**GLUT1**	AGCCCTGCTACAGTGTAT	AGGTCTCGGGTCACATC	26
**GLUT2**	TGTGCTGCTGGATAAATTCGCCTG	AACCATGAACCAAGGGATTGGACC	26
**GLUT5**	TCTTTGTGGTAGAGCTTTGGG	GACAATGACACAGACAATGCTG	26
**GLUT9**	TGCTTCCTCGTCTTCGCCACAATA	CTCTTGGCAAATGCCTGGCTGATT	26
**PGC1β**	TTCAGATGGAACCCCAAGC	ATCTCACCGAACACCTCAAAG	13

### Oil Red-O Staining

Methanol-fixed frozen sections from WT and GLUT8KO HFrD-fed mice were stained according to described protocols (28).

### Body Composition Analysis

Body composition analysis was carried out in unanesthetized mice as described (13) using an EchoMRI 3-1 device (Echo Medical Systems, Houston, TX) via the Washington University Diabetic Mouse Models Phenotyping Core Facility.

### Plasma and Hepatic Lipid Determination

Triglycerides and cholesterol were assayed using the Infinity triglycerides and cholesterol assay kits (Thermo Scientific, Rockford, IL) precisely according to the manufacturer's instructions. Free fatty acids were determined using the NEFA-HR(2) reagent kit (Wako Chemicals, Richmond, VA). Plasma was used directly in each assay. For hepatic tissue, lipids were extracted from ∼100 mg of hepatic tissue homogenized in 2:1 chloroform/methanol. 0.25–0.5% of each extract was evaporated for 1 h prior to biochemical determination per manufacturer's instructions.

### Indirect Calorimetry

Indirect calorimetry was performed in an Oxymax Indirect Calorimeter (Columbus Instruments, Columbus, OH) as described (13) via the Washington University Diabetic Mouse Model Phenotyping Core Facility.

### Statistical Analysis

Unless otherwise indicated, assays were performed a minimum of three times with similar results. Data are expressed as mean ± S.E. Data were analyzed using two-tailed homoscedastic T-tests with Bonferroni-Dunn post hoc correction when multiple comparisons were performed on data sets as indicated. *p* < 0.05 was defined as statistically significant.

## RESULTS

### 

#### 

##### GLUT8 mRNA Is Abundant in Murine and Human Liver Tissue

**FIGURE 1.**
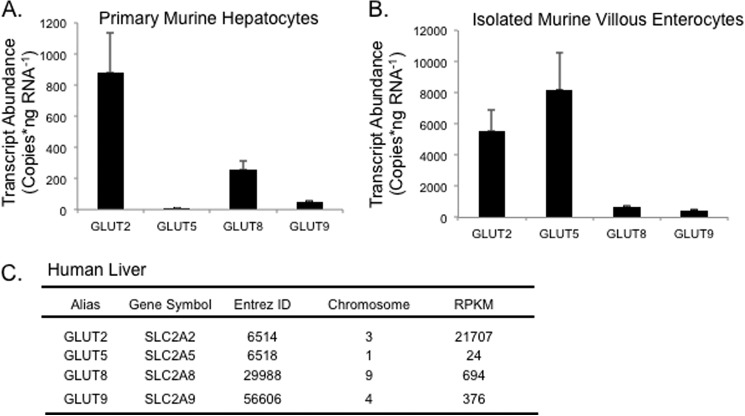
**Glucose transporter mRNA abundance in liver and enterocytes.** qRT-PCR quantification of GLUT2, GLUT5, GLUT8, and GLUT9 in primary mouse hepatocytes (*A*) and in isolated mouse enterocytes (*B*). *p* < 0.01 for all interactions by one-way analysis of variance and Tukey's honest significant difference post hoc correction. Data in *A* and *B* are from *n* = 7 wild-type mice, each run in triplicate. *C*, GLUT quantification in human liver tissue using RNA-seq data from the Illumina BodyMap project (version 2.0) as described under “Experimental Procedures.” Data are expressed as reads per kilobase per million mapped reads (*RPKM*).

The relative mRNA quantities of hepatic and enterocyte GLUTs with fructose transport capacity were first determined by real-time quantitative RT-PCR. GLUT2 transcript was most abundant in murine hepatocytes, followed by GLUT8 and GLUT9 (Fig. 1*A*). GLUT5 was least abundant of those transcripts studied (six copies/ng RNA). Conversely, evaluation of isolated murine villous enterocytes as a positive control for GLUT5 transcript demonstrated enterocyte GLUT5 and GLUT2 transcripts were most abundant, followed by GLUT8 and GLUT9 (Fig. 1*B*). Analysis of RNA-seq data from human liver demonstrated quantitative relationships similar to those in murine hepatocytes. GLUT2 was ∼30-fold more abundant than GLUT8 in human liver, whereas GLUT8 itself was greater than GLUT9 and GLUT5 in reads per kilobase per million reads mapped (Fig. 1*C*). These data suggest that GLUT8 is a relatively abundant glucose and fructose transporter in both murine and human liver.

##### GLUT8 Is a Plasma Membrane-localized Hepatocyte Fructose Transporter

**FIGURE 2.**
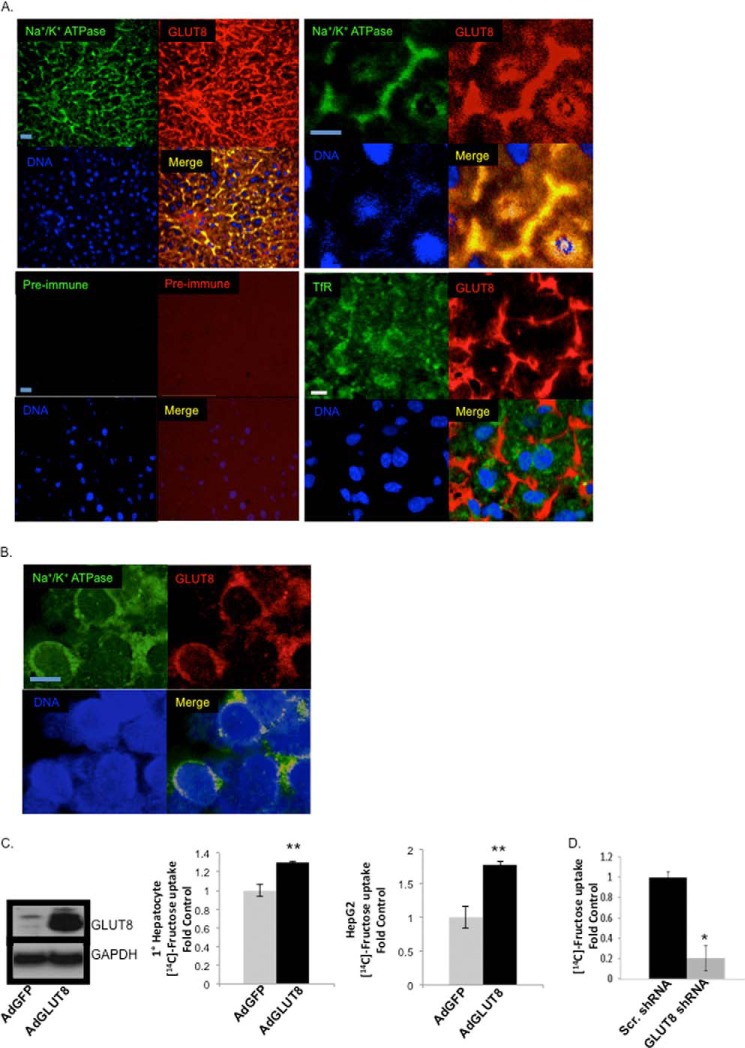
**GLUT8 is a rate-limiting, cell-surface hepatic fructose transporter.** *A*, immunofluorescence confocal microscopy in frozen liver sections derived from chow-fed, wild-type mice. *Upper panels*: low-power (*left*) and high-power (*right*) views of Na^+^/K^+^ ATPase α1 subunit (*green*), GLUT8 (*red*), and DNA (*blue*). *Lower panels*: *left*, confocal images following pre-immune primary serum and fluorescently labeled secondary antiserum, (*right*) transferrin receptor localization with GLUT8. *B*, immunofluorescence confocal microscopy in HepG2 cultures in standard growth medium (4.5 g/liter glucose). *Green*, Na^+^/K^+^ ATPase α1 subunit. *Red*, GLUT8. *Blue*, DNA. *Scale bars*, 10 μm. *C*, fructose uptake in primary mouse hepatocytes (*middle panel*) or in HepG2 cultures (*right panel*) transfected with adenovirus encoding green-fluorescent protein (*AdGFP*) or GLUT8 (*AdGLUT8*). **, *p* ≤ 0.01 *versus* AdGFP (*n* = 4–6). GLUT8 overexpression was confirmed by GLUT8 and GAPDH immunoblotting in primary hepatocytes transfected in parallel (*left panel*). *D*, fructose uptake in HepG2 cells transfected with lentivirus encoding scrambled (*Scr.*) or human GLUT8-directed shRNA. *, *p* < 0.05 *versus* scrambled (*n* = 4–6).

GLUT8 localization and function varies with the tissue in which it is expressed. For example, GLUT8 participates in insulin-stimulated cell-surface hexose transport in the blastocyst (23), whereas GLUT8 is primarily an intracellular hexose transporter in enterocytes (14). Because GLUT8-deficient mice exhibited impaired first-pass hepatic fructose metabolism after oral fructose gavage (14), we tested the hypothesis that GLUT8 localizes to the plasma membrane in murine hepatocytes by immunofluorescence confocal microscopy. GLUT8 co-localized with the Na^+^/K^+^ ATPase α1 subunit (Pearson correlation coefficient, 0.77 ± 0.05) to a significantly greater extent than with the predominantly endosomal transferrin receptor (Pearson correlation coefficient, 0.10 ± 0.6, *p* < 0.001 *versus* Na^+^/K^+^ ATPase α1 subunit) (Fig. 2*A*). GLUT8 and Na^+^/K^+^ ATPase α1 subunit co-localization was reproduced in HepG2 hepatoblastoma cells (Fig. 2*B*, Pearson correlation coefficient, 0.86 ± 0.03), whereas incubation of frozen liver sections with pre-immune sera did not produce measurable signal (Fig. 2*A*). Together, these data suggested hepatic GLUT8 is predominantly plasma membrane-localized (29).

GLUT8 localization at the hepatocyte plasma membrane prompted us to ask whether GLUT8 overexpression or knockdown could alter fructose accumulation rates *in vitro*. HepG2 and isolated primary hepatocyte cultures transfected with GLUT8-encoding adenovirus had ∼80 and 30% greater hepatocyte [^14^C]d-fructose uptake (Fig. 2C) in comparison with cultures transfected with control (*e.g.* GFP-encoding) adenovirus, respectively. Concordantly, GLUT8 gene silencing in HepG2 cultures transfected with lentivirus encoding GLUT8-directed short hairpin RNA blocked [^14^C]-d-fructose uptake by 80% compared with cultures transfected with scrambled shRNA-encoding lentivirus (Fig. 2*D*), suggesting that GLUT8 is a rate-limiting fructose transporter across the hepatocyte plasma membrane.

##### Diminished Fructose Uptake and de Novo Lipogenesis in GLUT8KO Hepatocytes

**FIGURE 3.**
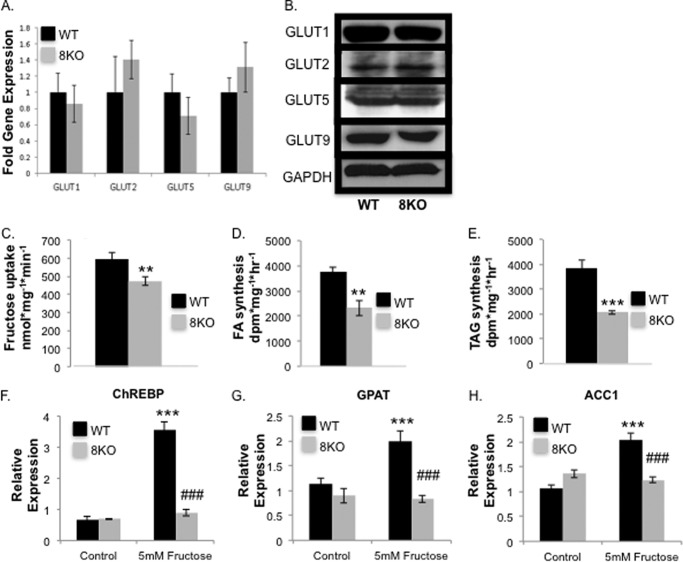
**GLUT8 required for fructose-induced *de novo* lipogenesis.** *A*, qRT-PCR of GLUT1, GLUT2, GLUT5, and GLUT9 in WT and GLUT8KO liver after 24 weeks HFrD (*n* = 4–11). *B*, GLUT1, GLUT2, GLUT5, and GLUT9 immunoblots in WT and GLUT8KO liver after 24 weeks of HFrD. *C*, decreased [^14^C]fructose uptake in cultured primary hepatocytes from chow-fed WT and GLUT8KO mice (*n* = 10 per group). *D* and *E*, decreased FA and triacylglycerol (*TAG*) synthesis from radiolabeled fructose in cultured hepatocytes from chow-fed WT and GLUT8KO mice (*n* = 4 per group). *F–H*, relative mRNA expression of fructose-inducible hepatocyte genes, carbohydrate response element binding protein (*ChREBP*), glycerol-3-phosphate acyltransferase (*GPAT*), acetyl coenzyme A carboxylase-1 (*ACC1*), analyzed by real-time qRT-PCR in the presence or absence of 5 mm fructose (24 h; *n* = 3–9 per group). **, *p* < 0.01 and ***, *p* < 0.001 *versus* WT controls. ###, *p* < 0.001 *versus* WT treated with 5 mm fructose.

Fructose delivery into the hepatocyte provides required substrates for fructose-induced *de novo* lipogenesis (1). The above data demonstrated that GLUT8 is required for maximal fructose delivery across the hepatocyte plasma membrane and that genetic GLUT8 ablation blocked fructose-induced steatosis *in vivo* without changes in hepatic fat oxidation markers or insulin signaling. We thus directly tested whether GLUT8 is required for hepatocyte fructose uptake and *de novo* lipogenesis by examining mice lacking GLUT8 (GLUT8KO mice). Analysis of liver tissue by real-time qRT-PCR analysis (Fig. 3*A*) and immunoblot analysis (Fig. 3*B*) from wild-type (WT) and GLUT8KO mice demonstrated unaltered mRNA and protein abundance of the other major hepatic hexose transporters, GLUT1, -2, -5, and -9. Hepatic GLUT12 protein and mRNA were not detectable in WT (30) or in GLUT8KO liver (data not shown). Nonetheless, isolated GLUT8KO hepatocytes exhibited significantly diminished fructose uptake (Fig. 3*C*). Downstream fatty acid (Fig. 3*D*) and triglyceride synthesis from fructose substrate (Fig. 3*E*) were concomitantly reduced in chow-fed GLUT8KO mouse hepatocytes. Consistent with diminished fructose entry and decreased *de novo* lipogenesis from fructose, fructose potently induced WT carbohydrate response element binding protein, glycerol-3-phosphate acyltransferase and acetyl coenzyme A carboxylase-1 (Fig. 3, *F–H*). In contrast, fructose failed to induce these genes in GLUT8KO hepatocytes.

**FIGURE 4.**
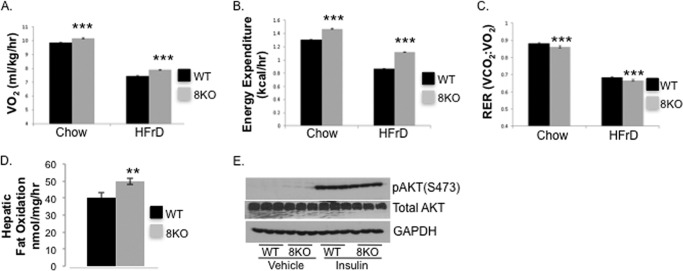
**Oxidative predilection without impaired proximal insulin signaling in GLUT8KO hepatocytes.** Oxygen consumption (*A*), caloric expenditure (*B*), and respiratory exchange ratio (*C*) in chow- and HFrD-fed WT and GLUT8KO mice (*n* = 7–9). *, *p* < 0.05 *versus* WT on equivalent diet. ***, *p* < 0.001 *versus* WT on equivalent diet. *D*, palmitate oxidation rates in cultured primary WT and GLUT8KO hepatocytes (*n* = 5). **, *p* ≤ 0.01 *versus* WT. *E*, immunoblot analysis of AKT phosphorylation (Ser-473) and GAPDH after insulin stimulation (500 nm, 5 min) in isolated hepatocytes from WT and GLUT8KO mice.

Together, the above findings indicated that GLUT8 is required for hepatocyte fructose uptake, fructose-induced *de novo* lipogenesis and lipogenic gene induction. Because the product of *de novo* lipogenesis, malonyl-CoA, blocks β-oxidation by inhibiting carnitine palmitoyltransferase I (31), we hypothesized that the attenuated *de novo* lipogenesis in GLUT8KO hepatocytes would result in greater β-oxdiation rates in GLUT8KO mice. We therefore assessed whether GLUT8 deficiency affected oxidative capacity both at the whole organism level and at the hepatocyte level. Indirect calorimetry in chow- or HFrD-fed WT and GLUT8KO mice revealed greater oxygen consumption (VO_2_) and caloric expenditure were higher in GLUT8KO mice *versus* WT controls at baseline and under HFrD-fed conditions (Fig. 4, *A* and *B*). Moreover, global substrate predilection in WT and GLUT8KO mice, as determined by the calculated ratio of carbon dioxide-to-oxygen consumption (respiratory exchange ratio), revealed a significantly lower respiratory exchange ratio in GLUT8KO mice after both chow and HFrD (Fig. 4*C*), consistent with a fatty acid oxidative predilection in GLUT8KO mice. Consistent with these findings, isolated primary GLUT8KO hepatocytes exhibited a significantly greater radiolabeled palmitate oxidation rate compared with WT hepatocytes *in vitro* (Fig. 4*D*). Nevertheless, qRT-PCR analysis of hepatic tissue from chow- and HFrD-fed WT and GLUT8KO mice demonstrated no significant changes in the predominant hepatic fatty acid oxidation regulatory transcription factors: LXR, FXR, and PPARα (data not shown). Moreover, GLUT8KO hepatocyte alterations in substrate predilection were not due to alterations in hepatic insulin-stimulated Akt Ser-473 phosphorylation, which was unaltered in GLUT8KO hepatocytes (Fig. 4*E*).

##### GLUT8 Is Required for Fructose-induced Hepatic Macrosteatosis

Given data suggesting that 1) GLUT8 is localized to the hepatocyte cell surface and 2) GLUT8 mediates hepatocyte fructose uptake, fructose-induced *de novo* lipogenesis, and lipogenic gene induction, we examined GLUT8-deficient (GLUT8KO) mice to determine whether GLUT8 mediates fructose-induced hepatic steatosis *in vivo*. GLUT8-deficient females were live-born with slightly decreased body weight and otherwise normal gross characteristics (13, 14, 20).

**FIGURE 5.**
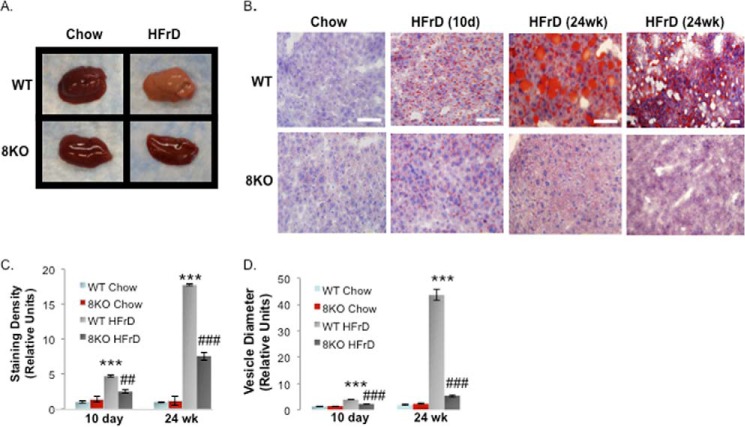
**Protection from fructose-induced hepatic steatosis in GLUT8KO mice.** *A*, gross WT and GLUT8KO livers after 10-day (*10d*) chow or HFrD. *B*, oil Red-O staining in chow- and HFrD-fed GLUT8KO and WT liver. *Scale bars*, 100 μm. Shown are oil Red-O staining density (*C*) and droplet diameters (*D*) in chow- and HFrD WT and GLUT8KO liver (*n* = 8–12 random fields from four to eight mice per group). ***, *p* < 0.001 *versus* chow-fed WT. ## and ###, < 0.01 and < 0.001 *versus* WT HFrD after post hoc correction, respectively.

To determine whether GLUT8 was required for HFrD-induced hepatic steatosis *in vivo*, WT and GLUT8KO mice were fed chow or HFrD for 10 days prior to sacrifice and dissection. Livers from HFrD-fed WT animals showed marked pallor on gross inspection, whereas livers from GLUT8KO mice on the same diet were grossly normal in appearance (Fig. 5*A*). Neutral lipid content assessment by Oil red-O staining in HFrD-fed WT and GLUT8KO frozen liver sections revealed significantly lower staining density in GLUT8KO livers *versus* WT controls after 10-day HFrD (Fig. 5*B*). After 24-week HFrD, WT livers became massively macrosteatotic with marked vacuolization, whereas GLUT8KO livers had reduced Oil Red-O staining at both 10 days and 24 weeks post-HFrD initiation (Fig. 5*B*). Quantification of Oil Red-O staining density demonstrated markedly increased staining density in WT mice *versus* chow-fed WT mice after both 10 days and 24 weeks of HFrD exposure (Fig. 5*C*). However, GLUT8KO livers exhibited significantly lower staining density (Fig. 5*C*) and smaller lipid droplet diameters (Fig. 5*D*) *versus* HFrD-fed WT livers following 10-day and 24-week HFrD. This suggested that the fructose transporter GLUT8 is essential to develop fructose-induced intrahepatic macrosteatosis.

##### GLUT8KO Mice Are Protected from Fructose-induced Lipid Accumulation

We previously showed GLUT8-deficient females on a long term HFrD had an exacerbated fructose-induced metabolic syndrome due in part to enhanced enterocyte fructose absorption (14). This enhanced metabolic syndrome included relative hypertension, hyperlipidemia, and hyperinsulinemia. Hyperinsulinemia is considered to be an exacerbating component in NAFLD pathogenesis, however. Thus, we performed additional hepatic analyses in intermediate term (4 weeks) HFrD-exposed WT and GLUT8KO animals, such that frank hyperinsulinemia was absent. Echo MRI body composition analysis revealed that 4-week HFrD-exposed GLUT8KO mice developed significantly greater body fat mass and lower lean mass percentages relative to WT controls (Table 2); yet fasting plasma insulin was identical (Table 2). Therefore, the subsequent GLUT8KO hepatic phenotypes occurred independently of changes in insulin homeostasis.

**TABLE 2:** **Physiologic and morphometric parameters in WT and GLUT8KO mice on 4-week chow or HFrD** LW, liver weight; BW, body weight.

	WT chow	8KO chow	WT HFrD	8KO HFrD
*n*	7	6	4	9
End weight (g)	19.5 ± 0.9	22.8*^a^* ± 0.3	24.1*^b^* ± 0.6	22.9 ± 0.7
Total water (g)	9.2 ± 0.4	11.1*^a^* ± 0.3	10.4 ± 0.5	9.7 ± 0.3
Fat mass (g)	6.5 ± 0.2	7.0 ± 0.2	8.2*^b^* ± 0.2	8.6 ± 0.4
Fat %	27.7 ± 1.3	30.5 ± 0.9	34.0*^b^* ± 0.3	37.5*^a^* ± 1.0
Lean mass (g)	13.9 ± 0.6	15.7*^a^* ± 0.3	15.6 ± 0.5	14.1*^a^* ± 0.4
Lean %	71.8 ± 1.3	69.0 ± 0.9	65.0*^b^* ± 0.4	61.7*^a^* ± 1.0
Liver mass (g)	591 ± 65	678 ± 18	839*^b^* ± 37	797 ± 42
LW/BW	29.5 ± 1.4	31.5 ± 0.5	36.5*^b^* ± 0.6	36.9 ± 1.7
Insulin (ng/ml)	1.4 ± 0.2	1.0 ± 0.1	1.5 ± 0.1	1.3 ± 0.2

*^a^ p* < 0.05, 8KO versus WT on equivalent diet.

*^b^ p* < 0.05 WT HFrD versus WT chow-fed.

**FIGURE 6.**
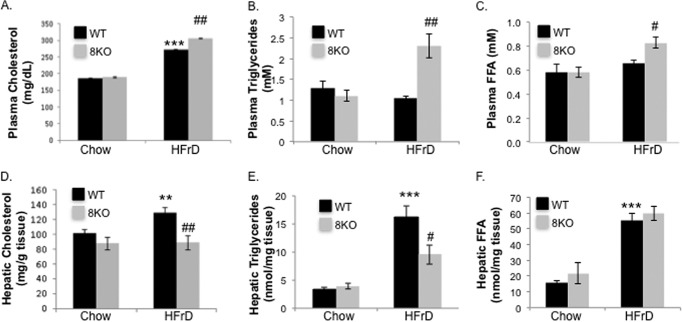
**Fasting plasma (*A–C*) and hepatic (*D–F*) lipids in chow- and HFrD-fed (4 weeks) WT and GLUT8KO mice.** *n* = 4–9 mice per group. **, *p* ≤ 0.01 and ***, *p* < 0.001 *versus* chow-fed WT. #, ##, and ###, *p* < 0.05, *p* < 0.01, and *p* < 0.001 *versus* WT HFrD after post hoc correction, respectively.

Hepatic analysis in 4-week HFrD-exposed mice revealed significant increases in both absolute (undessicated) liver mass and liver mass to body mass ratio in HFrD-fed WT mice *versus* chow-fed WT mice. In contrast, GLUT8KO liver mass did not significantly increase *versus* chow-fed GLUT8KO liver mass (Table 2). Fasting plasma cholesterol, triglycerides, and free fatty acids (FFA) were significantly elevated in 4-week HFrD-fed GLUT8KO mice *versus* WT controls (Fig. 6, *A–C*, respectively) despite statistically identical chow and HFrD food consumption rates (data not shown). Conversely, both hepatic cholesterol and triglycerides were significantly lower in 4-week HFrD-fed GLUT8KO mice (Fig. 6, *D* and *E*). Hepatic FFA content, which is primarily extrahepatically derived from adipose tissue lipase activity, was similar in GLUT8KO mice both under chow- and 4-week HFrD-fed conditions (Fig. 6*F*). Parallel with these changes in fat content, qRT-PCR analysis of hepatic tissue from HFrD-fed mice revealed significantly lower fatty acid synthease and sterol response element binding protein (fatty acid synthase and SREBP) mRNA abundance in GLUT8KO liver when compared with WT mouse liver (data not shown).

## DISCUSSION

Fructose is a potent steatogenic stimulus in the liver, largely due to metabolism of the six fructose carbons into glyceraldehyde-3-phosphate and dihydroxyacetone phosphate - both of which are triglyceride synthetic precursors (1). In addition to altered *de novo* lipogenesis, genes regulating a variety of processes are proposed to modify the development of NAFLD (reviewed in (5)). This includes genes regulating insulin resistance, hepatic lipid uptake and export, hepatic triglyceride hydrolysis, hepatic oxidative stress, fibrosis, and endotoxin (and other immune modulatory) responses. The proximal event in this complex pathogenic cascade, however, is fructose delivery into the hepatocyte.

Classically mediating fructose access to *de novo* lipogenic enzymes within the hepatocyte cytoplasm are the fructose transporters, GLUT2 (11) and GLUT5 (8). Further evidence demonstrated GLUT8 is expressed in murine liver and that hepatic GLUT8 expression is altered in both type 1 and type 2 diabetic mouse models (12). The physiological functions of GLUT8 in the liver, however, remained unclear. Here we show that GLUT8 is a plasma membrane-localized hepatocyte hexose transporter required for fructose-induced steatosis, a precursor in the NAFLD and non-alcoholic steatohepatitis spectrum. Moreover, isolated hepatocytes from chow-fed GLUT8KO mice exhibited basal defects in fructose transport and *de novo* lipogenesis in the absence of basal alterations in fatty acid synthase or SREBP1 mRNA. Fatty acid synthase and SREBP1 mRNA were diminished only after 10-day HFrD. This suggests at least two beneficial effects derived from blocking hepatocyte membrane fructose transport: 1) acutely diminished post-prandial fructose uptake, directly reducing substrate for conversion to fat in the post-prandial state, and 2) reduced chronic fructose entry into the cytosol, reducing the amplitude by which fructose can induce *de novo* lipogenic genes.

Hepatic GLUT8 deficiency may also confer protection from fructose-induced macrosteatosis through mechanisms other than impaired plasma membrane transport. First, GLUT8 was localized both at the plasma membrane and intracellularly here and in other studies (11–14). An intriguing possibility remains that GLUT8 also transports substrates between hepatic intracellular compartments. However, even short uptake times used herein (60 s in the primary hepatocyte uptake assays) indicate that GLUT8 affects very proximal levels of fructose uptake. Studies specifically addressing intracellular transport mechanisms of GLUT8 may yield further insight into its putative intracellular functions.

Second, GLUT8 appears to serve important basal functions in hepatic energy metabolism. Indeed, basal oxygen consumption, energy expenditure, and predilection to fat metabolism were elevated under chow- and fructose-fed conditions. Moreover, primary hepatocyte studies presented here were performed in cultures isolated from chow-fed mice prior to fructose or palmitate challenge. β-Oxidation rates and lipid synthesis rates therefore reflect intrinsic changes to GLUT8KO hepatocytes, raising the possibility that GLUT8KO mice could be protected from other hepatic steatotic stimuli, independent of fructose feeding.

Both GLUT8KO males (13) and females exhibited enhanced global thermogenesis and greater hepatocyte fatty acid oxidation, which we previously postulated to result from a relative hexose-deprived state in context of decreased hepatocyte hexose entry (13). Yet, only female GLUT8KO mice resist fructose-induced hepatic lipid accumulation. Although sexual dimorphism is seen in multiple diet-induced NAFLD models (*e.g.* in Toll-like receptor-4KO mice and in apolipoprotein E2 knock-in mice (31, 32)), the greater NAFLD resistance in our GLUT8KO female model is reconciled by data demonstrating abnormal fructose tolerance testing only in females (14). GLUT8 may thus participate in hepatic fructose entry from the enteric and portal circulation to a greater extent in females (13, 14). Under these circumstances, GLUT8KO females are predicted to be more resistant to diet-induced NAFLD than their male counterparts. Thus, we hypothesize that GLUT8KO female sex-specific hepatic portal fructose absorption defects with secondarily impaired *de novo* lipogenesis represent at least one key mechanism underlying GLUT8KO resistance to fructose-induced hepatic steatosis.

The HFrD-fed GLUT8KO model exhibited attenuated hepatic steatosis with an overall exacerbated metabolic syndrome. This finding is not without precedent; hepatic carbohydrate response element binding protein overexpression increased high-fat diet-induced hepatic steatosis while reducing circulating FFA levels, fasting insulin and glucose when compared with WT controls (33). However, the pathophysiology in that mouse model is similarly not fully apparent. In our model, decreased fructose transport and decreased *de novo* lipogenesis in GLUT8KO mice suggests the high circulating TG and cholesterol in HFrD-fed GLUT8KO mice comes from extrahepatic *de novo* lipogenic tissues such as enterocytes and adipocytes. Several pieces of data are consistent with shunting of oral fructose away from the GLUT8KO liver into these extrahepatic lipogenic pools: 1) increased enterocyte fructose uptake and peripheral fructose excursion after an oral bolus (14), 2) decreased hepatocyte fructose uptake, and 3) increased total adiposity in HFrD-fed GLUT8KO mice (Table 2). Because circulating FFA is more a reflection of total adipose mass in obese/insulin-resistant subjects (34), it follows that HFrD-fed GLUT8KO mice have higher circulating FFA to reflect the greater adipose mass in this group.

A potential limitation in the current study is the use of a traditional germ-line GLUT8 knock-out model in lieu of hepatocyte-specific GLUT8KO mice in studying HFrD-induced hepatic lipid accumulation. Although we cannot completely rule out extrahepatic protective mechanisms (*e.g.* alterations in inflammatory status, insulin, and glucose homeostasis, enterocyte fructose absorption) in germ line GLUT8KO mice, several observations indicate that extrahepatic protective mechanisms are unlikely to fully explain the observed phenotype. First, cell autonomously, attenuated fructose transport and fructose-stimulated *de novo* lipogenesis in isolated primary GLUT8KO hepatocytes were observed. Second, circulating C-reactive protein after chow or HFrD feeding groups did not differ in WT or GLUT8KO mice (14), suggesting similar global inflammatory status in the presence or absence of GLUT8. Third, liver from HFrD-fed cohorts at 10 days and at 4 weeks were studied; time points at which fructose-induced alterations in insulin and glucose homeostasis are not yet observed. Fourth, 24-week HFrD-fed GLUT8KO mice were hyperinsulinemic and dyslipidemic compared with WT controls, suggesting the GLUT8KO extrahepatic milieu is more potently steatogenic. Fifth, basal and fructose-induced enterocyte fructose absorption in female GLUT8KO mice is greater than in WT mice. Given the latter two observations, resistance to fructose-induced macrosteatosis in our globally GLUT8-deficient model would likely underestimate any protection conferred by hepatocyte-specific GLUT8 deletion.

Blocking fructose transport into the hepatocyte via the GLUT8 hexose transporter may thus be a potential mechanism to prevent or treat fructose-induced hepatic steatosis. Further studies characterizing the hepatic zonal and sex-specific roles of GLUT8 and the other hepatic hexose transporters, GLUT2 and GLUT5, will further inform this potentially clinically relevant paradigm.

## Footnotes

2 The abbreviations used are:

NAFLD: non-alcoholic fatty liver disease
GLUT: glucose transporter
HFrD: high-fructose diet
HBSS: Hanks' balanced salt solution
SREBP: sterol response element binding protein
qRT-PCR: quantitative real-time RT-PCR.
